# Predictors of Pathological Complete Response and Patient-Reported Outcomes During Neoadjuvant Chemotherapy in Early Breast Cancer: A Single-Center Retrospective Cohort Study

**DOI:** 10.7759/cureus.109160

**Published:** 2026-05-18

**Authors:** Isabela Anda Komporaly, Adelina Silvana Gheorghe, Elena Adriana Iovanescu, Lidia Anca Kajanto, Bogdan Georgescu, Dana Stanculeanu

**Affiliations:** 1 Department of Oncology, “Carol Davila” University of Medicine and Pharmacy, Bucharest, ROU; 2 Department of Medical Oncology, Memorial Hospital, Bucharest, ROU; 3 Department of Medical Oncology I, “Prof. Dr. Alexandru Trestioreanu” Institute of Oncology, Bucharest, ROU; 4 Department of Oncology, Neolife Medical Center, Bucharest, ROU; 5 Department of Medical Oncology, “Carol Davila” University of Medicine and Pharmacy, Bucharest, ROU

**Keywords:** breast cancer, hads, neoadjuvant chemotherapy, pathological complete response, patient-reported outcomes, quality of life, real-world data, tumor-infiltrating lymphocytes

## Abstract

Background: Neoadjuvant chemotherapy (NAC) is the standard of care for biologically aggressive early breast cancer, and pathological complete response (pCR) is a robust surrogate of long-term outcome. Real-world cohorts integrating clinical, pathological, and patient-reported variables remain limited.

Methods: We retrospectively analyzed 149 consecutive women with stage I-III invasive breast cancer treated with NAC followed by curative surgery at Memorial Hospital, Bucharest, Romania. The primary endpoint was overall pathological complete response (pCR), defined as the absence of residual invasive disease in both breast and axillary lymph nodes (ypT0/Tis ypN0; residual ductal carcinoma in situ permitted). Clinicopathological variables, NAC regimen, residual cancer burden (RCB), stromal tumor-infiltrating lymphocytes (TILs), and patient-reported outcomes (European Organisation for Research and Treatment of Cancer {EORTC} global health, Hospital Anxiety and Depression Scale {HADS}) at baseline and end of NAC were analyzed. Predictors of pCR were identified by univariate analysis and multivariable logistic regression with internal bootstrap validation.

Results: Median age was 54 years (IQR: 46-62). Subtype distribution was luminal B 68/149 (45.6%), human epidermal growth factor receptor 2 (HER2)-positive 49/149 (32.9%), and triple-negative 32/149 (21.5%). Overall, pCR was achieved in 41/149 patients (27.5%) as follows: 6/68 (8.8%) in luminal B, 26/49 (53.1%) in HER2-positive, and 9/32 (28.1%) in triple-negative breast cancer (TNBC) (χ²=27.95, df=2, p<0.001). pCR rates also differed markedly across NAC regimens (χ²=27.55, df=6, p<0.001), ranging from 1/17 (5.9%) for TC to 12/19 (63.2%) for taxane/trastuzumab/pertuzumab (THP). On multivariable analysis, clinical subtype, stromal TILs, and clinical nodal status were the principal independent predictors of pCR; the integrated model achieved an area under the receiver operating characteristic curve (AUC) of 0.93 (bootstrap-corrected: 0.92), with adequate calibration (Hosmer-Lemeshow χ²=4.90, df=6, p=0.557). EORTC global health declined by a median of four points between baseline and end of NAC (Wilcoxon W=2615.5, p<0.001), with 51/149 patients (34.2%) experiencing a clinically meaningful decline. At baseline, 30/149 patients (20.1%) had clinically significant anxiety, and 57/149 (38.3%) had borderline depressive symptoms; clinical depression emerged de novo in 18/149 (12.1%) by the end of NAC. Baseline patient-reported outcomes did not significantly predict pCR after adjustment for biological variables (likelihood-ratio χ²=4.71, df=3, p=0.194).

Conclusions: In this Romanian single-center cohort, pCR was independently predicted by clinical subtype and a limited set of biological variables, in line with contemporary literature. Although baseline patient-reported outcomes did not improve prediction of pathological response, the high prevalence of pre-treatment anxiety and the emergence of clinical depression during NAC argue for systematic psychosocial screening as part of standard neoadjuvant care.

## Introduction

Neoadjuvant chemotherapy (NAC) has become the standard of care for the majority of patients with locally advanced or biologically aggressive early breast cancer [1,2]. Beyond enabling breast-conserving surgery, NAC offers the opportunity to assess in vivo chemosensitivity and to escalate or switch systemic therapy in patients with residual disease. The proportion of patients receiving systemic therapy in the neoadjuvant rather than adjuvant setting has steadily increased over the past decade, and contemporary population-based analyses confirm that, when correctly indicated, neoadjuvant and adjuvant chemotherapy yield comparable long-term outcomes [3].

Pathological complete response (pCR), defined as the absence of invasive disease in breast and axillary lymph nodes (ypT0/Tis ypN0), is consistently associated with improved event-free and overall survival, particularly in human epidermal growth factor receptor 2 (HER2)-positive and triple-negative breast cancer (TNBC) subtypes [4,5]. The strength of this association has been reaffirmed in updated systematic reviews and meta-analyses of real-world cohorts, which support pCR as a clinically meaningful surrogate endpoint and a basis for risk-adapted post-neoadjuvant therapy [6]. Conversely, the prognostic relevance of pCR in hormone receptor-positive, HER2-negative (luminal B-like) tumors is more limited, although the achievement of pCR remains a favorable prognostic indicator within this subset [7,8].

Predicting pCR before or early during NAC has therefore become a central translational question. Established predictors include intrinsic subtype, hormone receptor status, HER2 status, Ki-67 proliferation index, clinical T and N category, and chosen NAC regimen [9,10]. More recently, stromal tumor-infiltrating lymphocytes (TILs), assessed on hematoxylin-eosin sections according to the International Immuno-Oncology Biomarker Working Group recommendations [11], have emerged as a robust biological predictor of response, particularly in TNBC and HER2-positive disease [12]. The relative weight of each of these variables in real-world, single-center cohorts continues to be of practical interest, especially where access to comprehensive molecular profiling is limited.

Beyond the biological dimension, NAC is a physically and psychologically demanding experience. Patients must integrate a recent cancer diagnosis, undergo prolonged treatment with significant toxicity, and live with diagnostic uncertainty regarding response. Patient-reported outcomes (PROs), including health-related quality of life (HRQoL) assessed by the European Organisation for Research and Treatment of Cancer Quality of Life Questionnaire - Core 30 (EORTC QLQ-C30) questionnaire and validated instruments for anxiety and depressive symptoms, such as the Hospital Anxiety and Depression Scale (HADS) [13,14], are increasingly recognized as both clinically meaningful endpoints and potentially modifiable factors that may influence treatment adherence and longer-term outcomes [15,16]. Whether baseline psychological distress carries independent prognostic information for pCR has been examined in only a small number of studies, with inconsistent results, and most published cohorts have not integrated PRO data alongside biological variables in the same prediction framework.

In this single-center retrospective study, we (i) describe the clinical, pathological, and patient-reported characteristics of an unselected NAC cohort treated at an oncology center in Bucharest, Romania; (ii) identify independent predictors of pCR among clinical subtype, pathological variables, NAC regimen, and stromal TILs; (iii) characterize changes in HRQoL and HADS scores from diagnosis to end of NAC; and (iv) evaluate whether baseline PROs add predictive information beyond biological variables.

## Materials and methods

Study design and population

We performed a retrospective cohort study of consecutive adult women with newly diagnosed, histologically confirmed invasive breast cancer (clinical stages I-III) who received NAC followed by curative-intent surgery at Memorial Hospital, an oncology center in Bucharest, Romania, between January 2024 and December 2025. Patients with metastatic disease at diagnosis (cM1), inflammatory breast cancer, prior contralateral breast cancer treatment, or incomplete surgical or pathological data were excluded. This study was approved by the institutional ethics committee and conducted in accordance with the Declaration of Helsinki and Good Clinical Practice. Given the retrospective design and the use of fully anonymized data, the requirement for individual informed consent was waived.

Clinicopathological assessment

Clinical staging was based on physical examination and breast imaging (ultrasound, mammography, and/or magnetic resonance imaging), in line with the Eighth Edition of the American Joint Committee on Cancer (AJCC) staging system [17]. Pre-NAC pathology was performed on core-needle biopsies and included histological subtype, grade, estrogen receptor (ER), and progesterone receptor (PR) by immunohistochemistry, HER2 status (immunohistochemistry with reflex in situ hybridization for equivocal cases), and Ki-67 proliferation index. Clinical subtype was assigned as luminal B (HR-positive/HER2-negative), HER2-positive (regardless of HR status), or TNBC (ER<1%, PR<1%, HER2-negative). Stromal TILs were scored on diagnostic biopsy specimens as a continuous variable (percentage of stromal area infiltrated by mononuclear inflammatory cells), strictly following the methodology established by the International Immuno-Oncology Biomarker Working Group [11].

Neoadjuvant therapy and surgery

NAC regimens were selected according to subtype and contemporaneous institutional practice and included anthracycline-taxane sequences (AC-T, dose-dense AC-T), platinum-containing combinations for TNBC (carboplatin/taxane → AC), HER2-directed regimens combining taxane with single or dual HER2 blockade where indicated (taxane/trastuzumab/pertuzumab {THP}, doxorubicin/cyclophosphamide followed by taxane/trastuzumab/pertuzumab {AC-THP}, docetaxel/cyclophosphamide plus trastuzumab/pertuzumab {TCHP}), and TC for selected patients. Relative dose intensity, dose reductions, treatment delays, and grade ≥3 toxicities were recorded. Surgery (breast-conserving surgery or mastectomy with sentinel lymph node biopsy, targeted axillary dissection, or axillary lymph node dissection as indicated) was performed within two to six weeks of NAC completion.

Endpoints and pathological response

The primary endpoint was overall pCR, defined as the absence of residual invasive disease in both the breast and axillary lymph nodes (ypT0/Tis ypN0), with residual ductal carcinoma in situ permitted. This definition is distinct from breast-only pCR (ypT0/Tis, irrespective of nodal status) and from the more stringent ypT0 ypN0 definition (which excludes residual in situ disease); throughout this manuscript, the term "pCR" refers to overall pCR (ypT0/Tis ypN0) unless otherwise specified. Secondary endpoints included breast pCR (ypT0/Tis), nodal pCR (ypN0), and residual cancer burden (RCB) class (0-III) computed using the algorithm originally described by Symmans et al., with the standard MD Anderson web-based calculator (Houston, TX: The University of Texas MD Anderson Cancer Center) [18].

Patient-reported outcomes

Patient-reported outcomes were collected as part of routine institutional practice at the following two time points: within four weeks before NAC initiation (baseline) and within four weeks after NAC completion/before surgery (end of NAC). The EORTC QLQ-C30 Global Health Status/quality of life subscale, linearly transformed to a 0-100 scale (higher scores indicating better health), was used for health-related quality of life (HRQoL) [13]. The Hospital Anxiety and Depression Scale (HADS), originally described by Zigmond and Snaith [14], was administered to assess anxiety (HADS-A) and depressive (HADS-D) symptoms; each subscale ranges from 0 to 21, with established cut-offs of 0-7 (normal), 8-10 (borderline), and 11-21 (clinically significant) [14,19]. A clinically meaningful HRQoL change was pre-defined as a 10-point difference, in line with EORTC interpretation guidance for the global health subscale [20].

Statistical analysis

Continuous variables were summarized as median (IQR) and compared between groups using the Mann-Whitney U test. Categorical variables were summarized as n (%) and compared using the Pearson chi-square test; Fisher's exact test was used when expected cell counts were <5. Paired changes in patient-reported outcomes (PROs) were compared using the Wilcoxon signed-rank test; changes across subtypes were compared using the Kruskal-Wallis test.

Predictors of pCR were first evaluated in univariate logistic regression. Variables with p<0.10 in univariate analysis or with a strong a priori biological rationale were entered into a multivariable model. Clinical subtype was entered with luminal B as reference. Discrimination was quantified using the area under the receiver operating characteristic curve (AUC). Internal validation used 500 bootstrap iterations to estimate optimism and produce a bias-corrected AUC, following the procedure described by Harrell Jr et al. [21]. Calibration was assessed using the Hosmer-Lemeshow goodness-of-fit test [22]. Likelihood-ratio testing was used to compare nested models with and without PRO variables. All p-values were two-sided; statistical significance was set at p<0.05. For each test reported, both the test statistic value (χ², U, W, H, or likelihood-ratio χ² as appropriate) and the corresponding p-value are presented. Analyses were performed using Python 3.12 (Wilmington, DE: Python Software Foundation) with the scipy and scikit-learn libraries. No imputation was performed; missing data were addressed by complete-case analysis.

## Results

Cohort characteristics

A total of 149 patients met the inclusion criteria. Baseline characteristics are summarized in Table 1. The median age was 54 years (IQR: 46-62), and the median BMI was 26.0 kg/m² (IQR: 22.6-27.9). Most tumors were cT2 (110/149, 73.8%), node-positive (cN1-N2 in 110/149, 73.8%), and high-grade (G3 in 79/149, 53.0%). The dominant histology was invasive ductal carcinoma of no special type (90/149, 60.4%); mixed ductal/lobular carcinoma was present in 35/149 (23.5%), and pure invasive lobular carcinoma in 16/149 (10.7%). By clinical subtype, 68/149 patients (45.6%) had luminal B, 49/149 (32.9%) HER2-positive, and 32/149 (21.5%) triple-negative (TNBC) disease.

**Table 1:** Baseline clinicopathological characteristics of the cohort. Data are presented as n (%) for categorical variables and as median (IQR) for continuous variables. AC: doxorubicin/cyclophosphamide; AC-T: doxorubicin/cyclophosphamide followed by taxane; AC-THP: AC followed by taxane/trastuzumab/pertuzumab; Carbo: carboplatin; ddAC-T: dose-dense AC-T; ECOG: Eastern Cooperative Oncology Group; EORTC: European Organisation for Research and Treatment of Cancer; ER: estrogen receptor; HADS-A: Hospital Anxiety and Depression Scale - Anxiety subscale; HADS-D: HADS - Depression subscale; HER2: human epidermal growth factor receptor 2; NAC: neoadjuvant chemotherapy; NST: no special type; PR: progesterone receptor; TC: docetaxel/cyclophosphamide; TCHP: TC plus trastuzumab/pertuzumab; THP: taxane/trastuzumab/pertuzumab; TILs: tumor-infiltrating lymphocytes; TNBC: triple-negative breast cancer

Characteristics	Value (n=149)
Age (years), median (IQR)	54 (46-62)
BMI (kg/m²), median (IQR)	26.0 (22.6-27.9)
ECOG performance status, n (%)	
0	85 (57.0)
1	61 (40.9)
2	3 (2.0)
Smoking status, n (%)	
Never	54 (36.2)
Former	30 (20.1)
Current	36 (24.2)
Unknown	29 (19.5)
Histology, n (%)	
Invasive ductal (NST)	90 (60.4)
Mixed ductal/lobular	35 (23.5)
Invasive lobular	16 (10.7)
Metaplastic	8 (5.4)
Histological grade, n (%)	
Grade 2	70 (47.0)
Grade 3	79 (53.0)
Clinical T stage, n (%)	
cT1	21 (14.1)
cT2	110 (73.8)
cT3	18 (12.1)
Clinical N stage, n (%)	
cN0	39 (26.2)
cN1	62 (41.6)
cN2	48 (32.2)
Tumor size pre-NAC (mm), median (IQR)	35 (27-45)
Ki-67 pre-NAC (%), median (IQR)	51 (36-61)
ER expression (%), median (IQR)	20 (0-70)
PR expression (%), median (IQR)	0 (0-45)
Stromal TILs (%), median (IQR)	27 (15-42)
Clinical subtype, n (%)	
Luminal B	68 (45.6)
HER2-positive	49 (32.9)
Triple-negative (TNBC)	32 (21.5)
NAC regimen, n (%)	
ddAC-T	31 (20.8)
Carbo/taxane → AC	31 (20.8)
AC-THP	22 (14.8)
AC-T	21 (14.1)
THP	19 (12.8)
TC	17 (11.4)
TCHP	8 (5.4)
Cycles received, median (IQR)	8 (4-8)
Relative dose intensity (%), median (IQR)	90 (80-100)
Dose reduction, n (%)	80 (53.7)
Treatment delays, n (%)	61 (40.9)
Surgery type, n (%)	
Breast-conserving surgery	83 (55.7)
Mastectomy	66 (44.3)
EORTC global health baseline, median (IQR)	68 (58-77)
HADS-A baseline, median (IQR)	8 (5-10)
HADS-D baseline, median (IQR)	6 (4-8)

Neoadjuvant chemotherapy regimens were assigned by subtype. Anthracycline-taxane sequences were used in 52/149 patients (34.9%), comprising AC-T in 21/149 (14.1%) and dose-dense AC-T in 31/149 (20.8%). Carboplatin-taxane → AC sequences were used in 31/149 patients (20.8%), mainly in TNBC. HER2-directed regimens with single or dual HER2 blockade were administered in 49/149 (32.9%) patients as follows: AC-THP in 22/149 (14.8%), THP in 19/149 (12.8%), and TCHP in 8/149 (5.4%). The remaining 17/149 (11.4%) patients received TC. The median number of cycles received was 8 (IQR: 4-8), with a median relative dose intensity of 90% (IQR: 80-100). Dose reductions were required in 80/149 patients (53.7%) and treatment delays in 61/149 (40.9%). Surgery consisted of breast-conserving surgery in 83/149 patients (55.7%) and mastectomy in 66/149 (44.3%); the type of surgery did not differ by pCR status (Fisher's exact test, p=0.715).

Pathological complete response

**Figure 1:**
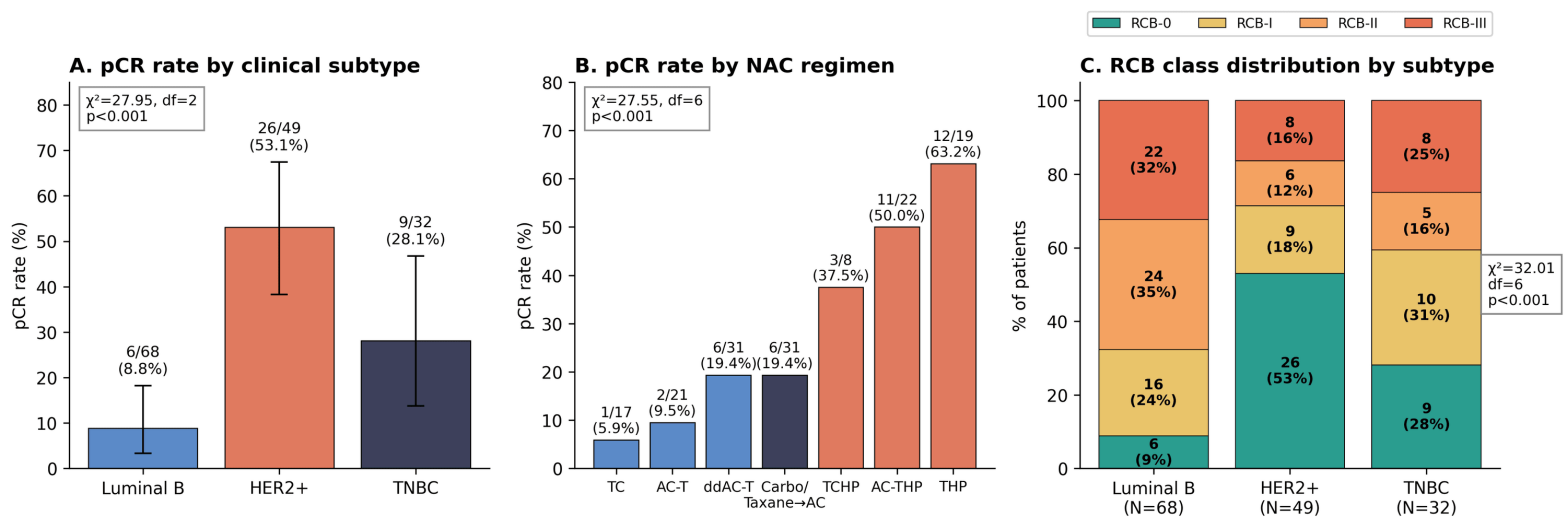
Pathological response by subtype, NAC regimen, and residual cancer burden distribution. Data are presented as n (%) above each bar in panels A and B. (A) pCR rate by clinical subtype with 95% confidence intervals (Wilson method); Pearson's chi-square test for subtype × pCR: χ²=27.95, df=2, p<0.001 (significance threshold p<0.05). (B) pCR rate by NAC regimen, ordered by ascending pCR rate; bars are colored according to the predominant clinical subtype receiving each regimen (blue=luminal B, orange=HER2-positive, and dark=TNBC). Pearson chi-square test for regimen × pCR: χ²=27.55, df=6, p<0.001. (C) Stacked bars showing the distribution of residual cancer burden classes (RCB-0 to RCB-III), computed using the algorithm of Symmans et al., within each clinical subtype, expressed as % [18]. pCR: pathological complete response; NAC: neoadjuvant chemotherapy; RCB: residual cancer burden; HER2: human epidermal growth factor receptor 2; TNBC: triple-negative breast cancer; AC: doxorubicin/cyclophosphamide; TC: docetaxel/cyclophosphamide; AC-T: doxorubicin/cyclophosphamide followed by taxane; AC-THP: AC followed by taxane/trastuzumab/pertuzumab; Carbo: carboplatin; ddAC-T: dose-dense AC-T; TCHP: TC plus trastuzumab/pertuzumab; THP: taxane/trastuzumab/pertuzumab

Overall, pCR was achieved in 41/149 patients (27.5%; 95% CI: 20.5-35.4). The pCR rates differed markedly across clinical subtypes: 6/68 (8.8%) in luminal B, 26/49 (53.1%) in HER2-positive, and 9/32 (28.1%) in TNBC (χ²=27.95, df=2, p<0.001) (Figure 1A). The pCR rates also varied substantially across NAC regimens (χ²=27.55, df=6, p<0.001), from 1/17 (5.9%) for TC and 2/21 (9.5%) for AC-T at the lower end to 11/22 (50.0%) for AC-THP and 12/19 (63.2%) for THP at the upper end (Figure 1B). This gradient mirrored subtype-specific prescribing as follows: TC and standard AC-T were used mainly in HR-positive disease; dose-dense AC-T and carboplatin-taxane → AC were used in higher-risk disease and TNBC; and HER2-directed regimens were used exclusively in HER2-positive disease. The distribution of residual cancer burden (RCB) classes by subtype is shown in Figure 1C and Table 2. RCB-0 was most frequent in HER2-positive disease (26/49, 53.1%), followed by TNBC (9/32, 28.1%) and luminal B (6/68, 8.8%). Conversely, RCB-II and RCB-III together accounted for 67.6% (46/68) of luminal B tumors but only 28.6% (14/49) of HER2-positive tumors, consistent with the relative chemoresistance of luminal B disease.

**Table 2:** Pathological response and residual cancer burden distribution by clinical subtype. Data are presented as n (%). Pathological complete response (pCR) (ypT0/Tis ypN0), residual cancer burden (RCB) class, computed using the algorithm of Symmans et al. [18]. Comparison of pCR proportions across subtypes by Pearson's chi-square test=27.95, df=2, p<0.001 (significance threshold p<0.05). Comparison of RCB distribution across subtypes by Pearson's chi-square test=46.4, df=6, p<0.001. HER2: human epidermal growth factor receptor 2; TNBC: triple-negative breast cancer

Subtype (n)	pCR, n (%)	RCB-0, n (%)	RCB-I, n (%)	RCB-II, n (%)	RCB-III, n (%)
Luminal B (68)	6 (8.8)	6 (8.8)	16 (23.5)	24 (35.3)	22 (32.4)
HER2-positive (49)	26 (53.1)	26 (53.1)	9 (18.4)	6 (12.2)	8 (16.3)
TNBC (32)	9 (28.1)	9 (28.1)	10 (31.2)	5 (15.6)	8 (25.0)
Total (149)	41 (27.5)	41 (27.5)	35 (23.5)	35 (23.5)	38 (25.5)

Univariate and multivariable predictors of pCR

In univariate analysis, the variables significantly associated with pCR were clinical subtype (χ²=27.95, df=2, p<0.001), NAC regimen (χ²=27.55, df=6, p<0.001), ER expression (lower in patients with pCR; Mann-Whitney U=1522.0, p=0.002), PR expression (also lower in patients with pCR; U=1556.5, p=0.003), and stromal TILs (median: 47% in pCR vs. 20.5% in non-pCR; U=3927.0, p<0.001) (Table 3). Tumor size, Ki-67 index, grade, cT and cN category, age, BMI, and ECOG performance status were not significantly associated with pCR. As expected, the median stromal TILs varied by subtype, with the highest values in TNBC (median: 47%, IQR: 30-58) and HER2-positive disease (median: 37%, IQR: 31-48), and the lowest in luminal B (median: 17%, IQR: 9-23).

**Table 3:** Univariate predictors of pathological complete response. ^*^Statistical significance was set at p<0.05. Data are presented as n (%) for categorical variables and as median (interquartile range {IQR}) for continuous variables. Continuous variables compared using the Mann-Whitney U test; categorical variables compared using the Pearson's chi-square test (or Fisher's exact test where appropriate). Test statistic shown as U for Mann-Whitney, χ² for chi-square (with degrees of freedom, df). pCR: pathological complete response; NAC: neoadjuvant chemotherapy; HER2: human epidermal growth factor receptor 2; TNBC: triple-negative breast cancer; AC: doxorubicin/cyclophosphamide; TILs: tumor-infiltrating lymphocytes; ER: estrogen receptor; PR: progesterone receptor; ECOG: Eastern Cooperative Oncology Group; EORTC: European Organisation for Research and Treatment of Cancer; HADS-A: Hospital Anxiety and Depression Scale - Anxiety subscale; HADS-D: HADS - Depression subscale

Variables	Non-pCR (n=108)	pCR (n=41)	Test statistic	p-Value
Age (years), median (IQR)	54.5 (46.0-61.2)	54.0 (47.0-62.0)	U=2137.0	0.745
BMI (kg/m²), median (IQR)	25.6 (22.4-27.8)	26.3 (23.6-28.4)	U=2537.5	0.170
Tumor size pre-NAC (mm), median (IQR)	35 (27-44)	36 (30-49)	U=2482.0	0.255
Ki-67 pre-NAC (%), median (IQR)	50 (36-60)	55 (42-65)	U=2525.0	0.187
ER expression (%), median (IQR)	37.5 (0-75)	0 (0-45)	U=1522.0	0.002^*^
PR expression (%), median (IQR)	20 (0-50)	0 (0-0)	U=1556.5	0.003^*^
Stromal TILs (%), median (IQR)	20.5 (10.8-30.0)	47.0 (35-66)	U=3927.0	<0.001^*^
Clinical subtype, n (%)				
Luminal B	62 (57.4)	6 (14.6)	χ²=27.95, df=2	<0.001^*^
HER2-positive	23 (21.3)	26 (63.4)		
TNBC	23 (21.3)	9 (22.0)		
NAC regimen, n (%)	-	-	χ²=27.55, df=6	<0.001^*^
cT category, n (%)	-	-	χ²=2.97, df=2	0.227
cT1	16 (14.8)	5 (12.2)	-	-
cT2	82 (75.9)	28 (68.3)	-	-
cT3	10 (9.3)	8 (19.5)	-	-
cN category, n (%)				
cN0	27 (25.0)	12 (29.3)	χ²=1.59, df=2	0.453
cN1	43 (39.8)	19 (46.3)		
cN2	38 (35.2)	10 (24.4)		
Histological grade, n (%)				
Grade 2	54 (50.0)	16 (39.0)	χ²=1.03, df=1	0.272
Grade 3	54 (50.0)	25 (61.0)		
ECOG, n (%)				
0	60 (55.6)	25 (61.0)	χ²=3.10, df=2	0.213
1	47 (43.5)	14 (34.1)		
2	1 (0.9)	2 (4.9)		
EORTC global health baseline, median (IQR)	68.5 (58-77)	63 (55-77)	U=2009.0	0.384
HADS-A baseline, median (IQR)	8 (5-10)	7 (5-10)	U=2104.5	0.641
HADS-D baseline, median (IQR)	6 (4-8.2)	6 (3-9)	U=2128.5	0.716

**Figure 2:**
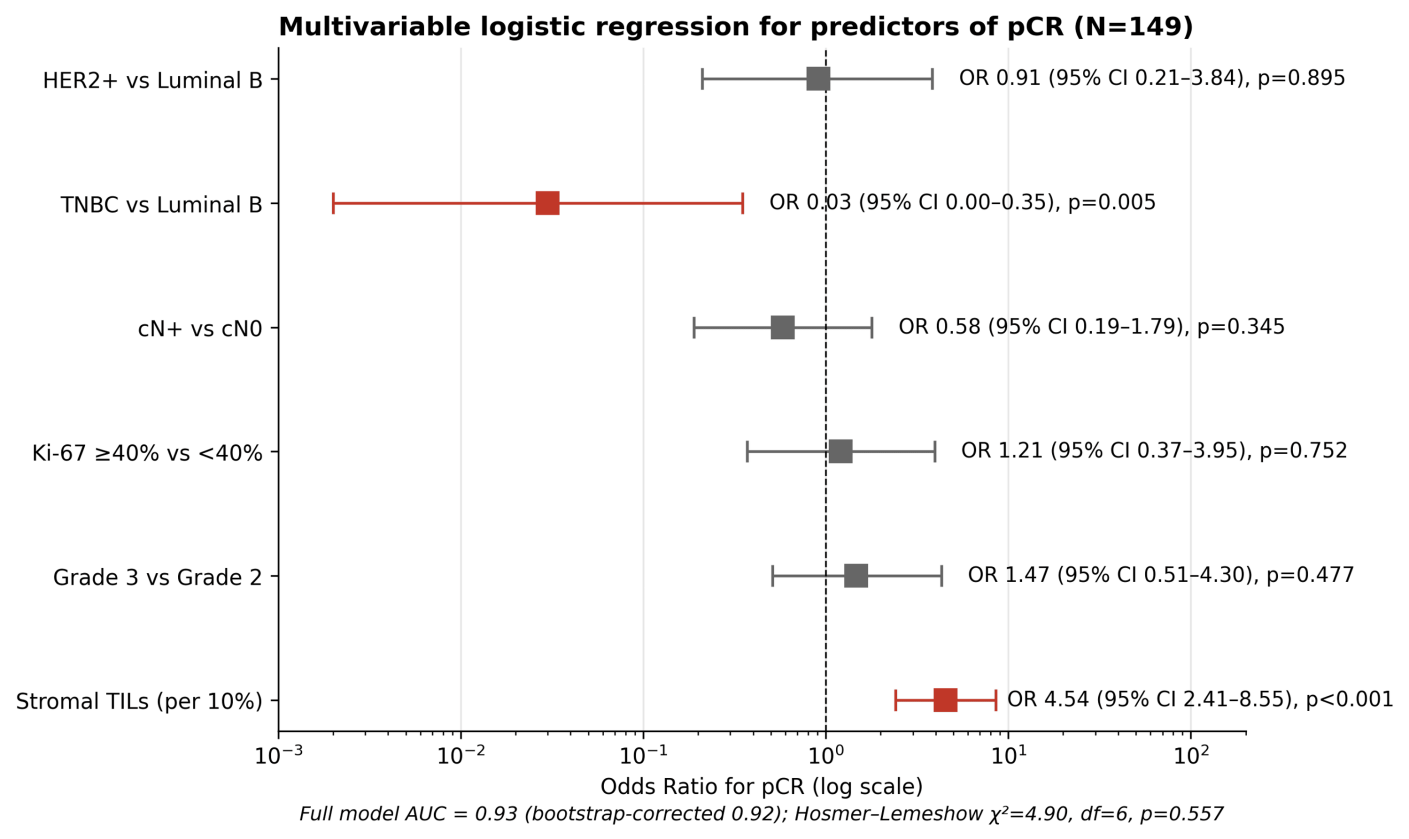
Multivariable logistic regression - independent predictors of pCR. Forest plot showing odds ratios (squares) and 95% confidence intervals (whiskers) for variables included in the multivariable logistic regression model. Reference categories - luminal B for subtype, cN0 for nodal status, Ki-67 <40% for proliferation, Grade 2 for histological grade. Odds ratio for stromal TILs is expressed per 10% increase. Statistically significant variables (95% CI excluding 1) are shown in red. The vertical dashed line at OR=1 indicates no effect. Significance threshold p<0.05. Full model discrimination: AUC=0.93 (bootstrap bias-corrected 0.92, 500 iterations [21]); calibration: Hosmer-Lemeshow χ²=4.90, df=6, p=0.557 [22]. AUC: area under the receiver operating characteristic curve; HER2: human epidermal growth factor receptor 2; TNBC: triple-negative breast cancer; pCR: pathological complete response; TIL: tumor-infiltrating lymphocytes

In the multivariable logistic regression model including clinical subtype, clinical nodal status, Ki-67 ≥40%, grade, and stromal TILs (modeled per 10% increase), three findings stood out. Clinical subtype remained associated with pCR, but its effect was attenuated compared with the univariate analysis as follows: the adjusted OR for TNBC vs. luminal B was 0.03 (95% CI: 0.002-0.35, p=0.005), while the HER2-positive vs. luminal B contrast was no longer significant after adjustment for TILs (adjusted OR: 0.91, 95% CI: 0.21-3.84, p=0.895). Ki-67, grade, and clinical nodal status were not independent predictors. Stromal TILs remained an independent predictor of pCR, with an adjusted OR of 4.54 per 10% increase (95% CI: 2.41-8.55, p<0.001) (Figure 2, Table 4). Model discrimination was high (apparent AUC: 0.93; bootstrap bias-corrected AUC: 0.92), and calibration was adequate (Hosmer-Lemeshow χ²=4.90, df=6, p=0.557). Adding stromal TILs to a clinical-only model (subtype, cN, Ki-67, grade) increased discrimination from AUC: 0.77 to AUC: 0.93 (likelihood-ratio χ²=56.14, df=1, p<0.001).

**Table 4:** Summary of statistical tests performed in this study. ^*^Statistical significance was set at p<0.05. All tests were two-sided. df: degrees of freedom; OR: odds ratio; ΔEORTC/ΔHADS: change between baseline and end of neoadjuvant chemotherapy; H: Kruskal-Wallis test statistic; U: Mann-Whitney U test statistic; W: Wilcoxon signed-rank test statistic; χ²: chi-square test statistic; pCR: pathological complete response; NAC: neoadjuvant chemotherapy; RCB: residual cancer burden; ER: estrogen receptor; PR: progesterone receptor; EORTC: European Organisation for Research and Treatment of Cancer; HADS-A: Hospital Anxiety and Depression Scale - Anxiety subscale; HADS-D: HADS - Depression subscale; QoL: quality of life; PRO: patient-reported outcome; TILs: tumor-infiltrating lymphocytes; BCS: breast-conserving surgery

Comparison/hypothesis tested	Test	Test statistic (df)	p-Value
pCR proportion across clinical subtypes	Pearson chi-square	χ²=27.95 (df=2)	<0.001^*^
pCR proportion across NAC regimens	Pearson chi-square	χ²=27.55 (df=6)	<0.001^*^
Surgery type (BCS vs. mastectomy) by pCR status	Fisher exact	OR=0.85	0.715
TILs distribution: pCR vs. non-pCR	Mann-Whitney U	U=3927.0	<0.001^*^
TILs distribution across RCB classes	Kruskal-Wallis	H=55.05 (df=3)	<0.001^*^
ER (%) distribution: pCR vs. non-pCR	Mann-Whitney U	U=1522.0	0.002^*^
PR (%) distribution: pCR vs. non-pCR	Mann-Whitney U	U=1556.5	0.003^*^
EORTC global health change (baseline → end of NAC)	Wilcoxon signed-rank	W=2615.5	<0.001^*^
HADS-A change (baseline → end of NAC)	Wilcoxon signed-rank	W=4386.0	0.995
HADS-D change (baseline → end of NAC)	Wilcoxon signed-rank	W=4064.0	0.877
ΔEORTC global health across subtypes	Kruskal-Wallis	H=0.39 (df=2)	0.823
ΔHADS-A across subtypes	Kruskal-Wallis	H=5.92 (df=2)	0.052
ΔHADS-D across subtypes	Kruskal-Wallis	H=0.46 (df=2)	0.793
Clinically meaningful QoL decline (≥10 points) across subtypes	Pearson chi-square	χ²=4.63 (df=2)	0.099
Multivariable model fit: TILs added to clinical-only model	Likelihood-ratio	χ²=56.14 (df=1)	<0.001^*^
Multivariable model fit: PRO variables added to biological	Likelihood-ratio	χ²=4.71 (df=3)	0.194
Calibration of final multivariable model	Hosmer-Lemeshow	χ²=4.90 (df=6)	0.557

Patient-reported outcomes at baseline and end of NAC

PRO data were available for all 149 patients at both time points. At baseline, the median EORTC global health score was 68 (IQR: 58-77), and the median HADS-A and HADS-D scores were 8 (IQR: 5-10) and 6 (IQR: 4-8), respectively. At the start of NAC, 30/149 patients (20.1%) already had clinically significant anxiety (HADS-A ≥11), and another 47/149 (31.5%) had borderline anxiety (HADS-A 8-10); for depression, 57/149 patients (38.3%) had borderline scores (HADS-D 8-10) and none reached the clinical threshold (Table 4).

**Figure 3:**
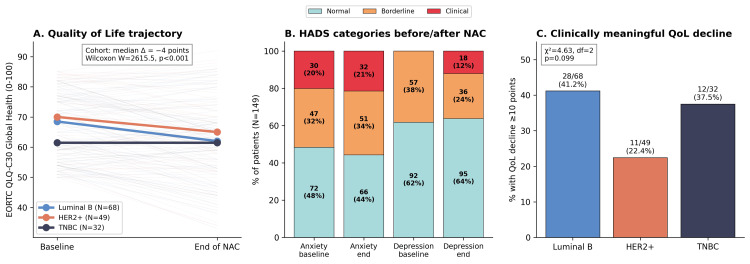
Patient-reported outcomes between baseline and end of neoadjuvant chemotherapy. (A) EORTC QLQ-C30 global health/quality of life score (0-100 scale, higher scores indicating better health) at baseline and at end of NAC; paired comparison by Wilcoxon signed-rank test, W=2615.5, p<0.001. The dashed reference line indicates the clinically meaningful change threshold of 10 points [20]. (B) Hospital Anxiety and Depression Scale subscale distributions (HADS-A, HADS-D) at baseline and end of NAC, stratified into normal (0-7), borderline (8-10), and clinically significant (11-21) categories; paired comparison by Wilcoxon signed-rank test: HADS-A W=4386.0, p=0.995; HADS-D W=4064.0, p=0.877. (C) Proportion of patients experiencing a clinically meaningful decline in EORTC global health (≥10 points) between baseline and end of NAC, stratified by clinical subtype; Pearson's chi-square test=4.63, df=2, p=0.099. Significance threshold p<0.05. EORTC: European Organisation for Research and Treatment of Cancer; HADS-A: Hospital Anxiety and Depression Scale - Anxiety subscale; HADS-D: HADS - Depression subscale; NAC: neoadjuvant chemotherapy; QoL: quality of life; HER2: human epidermal growth factor receptor 2; TNBC: triple-negative breast cancer

Across the cohort, the EORTC global health score declined significantly from baseline to end of NAC, with a median change of -4 points (IQR: -12 to +4; Wilcoxon W=2615.5, p<0.001) (Figure 3A). A clinically meaningful decline (≥10 points) occurred in 51/149 patients (34.2%) and was numerically more frequent in luminal B (28/68, 41.2%) and TNBC (12/32, 37.5%) than in HER2-positive disease (11/49, 22.4%; χ²=4.63, df=2, p=0.099). At the cohort level, the median HADS-A and HADS-D scores were unchanged (Wilcoxon W=4386.0, p=0.995 and W=4064.0, p=0.877, respectively). However, the proportion of patients meeting the threshold for clinically significant depression rose from 0/149 (0%) at baseline to 18/149 (12.1%) at the end of NAC (Figures 3B, 3C). A trend toward differential HADS-A trajectory across subtypes was observed (Kruskal-Wallis H=5.92, df=2, p=0.052), with the median HADS-A score increasing in HER2-positive patients (+1) and decreasing in luminal B patients (-1).

Incremental predictive value of baseline PROs

In univariate analysis, baseline EORTC global health (U=2009.0, p=0.384), HADS-A (U=2104.5, p=0.641), and HADS-D (U=2128.5, p=0.716) scores were not associated with pCR. When baseline PRO variables (EORTC global health <70, HADS-A ≥8, and age ≥50) were added to the biological multivariable model, the likelihood-ratio test was non-significant (χ²=4.71, df=3, p=0.194), showing that PROs at diagnosis did not add independent predictive information beyond clinical subtype and biological variables. A non-significant trend toward higher pCR odds in patients with low baseline global health was observed (adjusted OR: 2.89, 95% CI: 0.99-8.40, p=0.052); given the small number of events, this should be interpreted with caution. Changes in PROs between baseline and end of NAC did not differ significantly between patients who achieved pCR and those who did not (all p>0.50). A summary of all statistical tests performed in this study is provided in Table 4.

## Discussion

In this single-center retrospective cohort of 149 women treated with modern, subtype-tailored NAC at Memorial Hospital, Bucharest, we provide an integrated description of biological and patient-reported variables in relation to pCR. The overall pCR rate of 27.5% (41/149) and its distribution across subtypes (HER2-positive >> TNBC > luminal B) are consistent with pooled neoadjuvant analyses and recent real-world reports. In the CTNeoBC pooled analysis of more than 11,000 patients, pCR rates were lowest in HR-positive/HER2-negative grade 1-2 disease (~7%), intermediate in TNBC (~34%), and highest in HR-negative/HER2-positive disease treated with trastuzumab (~50%) [4]. Our subtype-specific rates fall within these ranges, with the modest gap in TNBC pCR likely reflecting heterogeneity in platinum exposure and the absence of routine immune checkpoint blockade during the study period. Real-world meta-analyses confirm the durable association between pCR and improved survival across multiple cohorts, including those treated outside large clinical trials [6].

Among biological predictors, clinical subtype and stromal TILs emerged as the dominant independent determinants of pCR in our model, whereas Ki-67 ≥40%, grade, and clinical nodal status did not remain significant after adjustment. The lack of independent contribution of Ki-67 in our cohort should be interpreted in the context of the high baseline proliferation of the cohort (median Ki-67 51%), which limits dynamic range. The strong, independent contribution of TILs aligns with the body of work establishing TILs as markers of immunogenicity and chemosensitivity in breast cancer and with recent recommendations to incorporate TIL assessment into routine pre-NAC pathology reporting in TNBC and HER2-positive disease [11,12]. The substantially lower adjusted odds of pCR in TNBC vs. luminal B after adjustment for TILs (adjusted OR: 0.03) are consistent with the interpretation that, within TNBC, the probability of pCR is strongly dependent on the magnitude of pre-existing immune infiltration as follows: TNBC tumors with low baseline TILs, the immunologically "cold" subset, appear to have a markedly lower probability of pCR than the cohort average would suggest, while TIL-rich TNBC tumors drive much of the apparent subtype-level pCR rate. The pooled individual-patient analysis by Loi et al., including more than 2,000 patients with early-stage TNBC, demonstrated that each 10% increase in stromal TILs was independently associated with improved invasive disease-free, distant disease-free, and overall survival; the magnitude of the effect we observed in our predictive model for pCR is concordant with the prognostic effect they reported for survival, supporting the biological continuity between immune infiltration, chemosensitivity, and long-term outcome [23].

The attenuation of the HER2-positive vs. luminal B contrast after adjustment for stromal TILs in our model should not be read as a refutation of the well-established subtype effect on pCR. One possible interpretation is that a portion of the apparent subtype effect operates through immune infiltration, which is more abundant in HER2-positive and TNBC tumors than in luminal B disease, and which is therefore partly captured by the TIL covariate. However, this interpretation must be advanced with caution as follows: the limited sample size, the strong collinearity between subtype and TILs in our cohort, the absence of HER2-directed therapy as a separate covariate, and the potential for residual confounding all preclude any firm inference about mediation. The pattern we observed is consistent with (but not proof of) a partially shared biological signal between subtype and immune infiltration, and requires confirmation in larger, multi-center cohorts.

The biological centrality of immune infiltration to neoadjuvant response has been further validated by the recent integration of immune checkpoint blockade into NAC protocols for high-risk early TNBC. The KEYNOTE-522 phase III trial demonstrated that the addition of pembrolizumab to neoadjuvant chemotherapy increased the pCR rate from 51.2% to 64.8% in early TNBC, with a corresponding improvement in event-free survival [24]. Similarly, the IMpassion031 trial showed that adding atezolizumab to sequential nab-paclitaxel and anthracycline-based chemotherapy improved pCR rates from 41% to 58% in early TNBC, particularly in the PD-L1-positive subgroup [25]. Our cohort, treated before the broad availability of immune checkpoint inhibitors in the neoadjuvant setting, may therefore represent a useful baseline against which the incremental impact of these agents could be assessed in future Romanian and Eastern European real-world cohorts. The strong predictive value of TILs we observed in TNBC also provides indirect support for the rationale of immune-based escalation in the immunologically "cold" subset of TNBC tumors with low baseline TILs, which represented a numerically substantial fraction of TNBC patients in our series.

A noteworthy feature of our cohort is the substantial heterogeneity in pCR across NAC regimens, ranging from 5.9% with TC to 63.2% with THP. While these differences predominantly reflect subtype-tailored prescription rather than intrinsic regimen efficacy, they illustrate how pCR rates in real-world cohorts must be interpreted within the framework of their underlying treatment patterns. International consensus on NAC has evolved from regimen-centric to subtype-centric prescription [2], a principle reaffirmed at the 2023 St Gallen International Consensus Conference and the 2024 ESMO Clinical Practice Guidelines for early breast cancer [10,26]. Our findings reinforce the practical importance of this principle as follows: regimen and subtype are inseparable in clinical practice, and meaningful comparisons require either subtype-stratified analysis or multivariable adjustment.

The relevance of pCR as an endpoint deserves particular attention in the context of long-term outcomes. Cortazar and Geyer Jr have emphasized that, while pCR is associated with markedly improved prognosis at the individual patient level, its utility as a regulatory surrogate at the trial level is most pronounced in HER2-positive and TNBC settings [5]. Beyond pCR as a binary endpoint, the long-term validation of the RCB index by Symmans et al., using a pooled cohort of more than 5,000 patients with extended follow-up, has firmly established that RCB class is independently associated with event-free and distant recurrence-free survival across all biological subtypes, including HR-positive/HER2-negative disease, where pCR alone has limited prognostic discrimination [27]. The clear gradient of stromal TILs across RCB classes that we observed (median 47% in RCB-0 down to 18-24% in RCB-I/II/III; H=55.05, df=3, p<0.001) supports the biological plausibility of TILs as a marker of integrated immunogenicity rather than a binary classifier, and is consistent with the broader notion that the depth of pathological response, not simply its presence or absence, captures meaningful clinical information.

The recent population-based analysis by Liu et al., drawing on more than 25,000 patients, further refined this picture by showing that long-term outcomes after neoadjuvant vs. adjuvant chemotherapy are comparable when stratified by biological subtype, and that the prognostic value of pCR is partially mediated by nodal therapy response [3]. The systematic review and meta-analysis by Antonini et al., restricted to real-world data, demonstrated a consistent and significant improvement in disease-free and overall survival among patients achieving pCR across heterogeneous practice settings, validating the pCR endpoint outside the controlled environment of registration trials [6]. Our cohort, while lacking mature survival data at the present time, falls within this real-world tradition and is expected to contribute follow-up evidence in due course.

A particularly relevant aspect of long-term outcome in our cohort concerns the luminal B subgroup, in which the majority of patients (46/68, 67.6%) had RCB-II/III residual disease, a population at substantial risk for late recurrence. The extended follow-up data assembled by the Early Breast Cancer Trialists' Collaborative Group, encompassing more than 62,000 women with HR-positive disease who completed five years of endocrine therapy, demonstrated that distant recurrence continues to accrue at a steady rate of 1-2% per year for at least 15 years thereafter, with cumulative 20-year distant recurrence rates ranging from 13% in node-negative low-volume disease to 41% in node-positive high-volume disease [28]. The Danish population-based analysis by Pedersen et al., extending observation up to 32 years from primary diagnosis, confirmed that recurrence risk in HR-positive breast cancer remains clinically significant well beyond the conventional follow-up window, with most very-late events occurring in patients who initially had a favorable response to treatment [29]. These observations underscore that pCR, and to a greater extent RCB, capture only part of the long-term risk landscape in HR-positive disease, and that the biology of tumor dormancy and minimal residual disease control over decades remains a central translational frontier. Whether the immune microenvironment, as captured by stromal TILs, modulates not only chemosensitivity but also the trajectory of dormant residual disease is a question of considerable interest that our planned long-term follow-up of this cohort will be positioned to address.

The most striking finding of our study lies not in the biological predictors but in the patient-reported domain. Even before any cytotoxic exposure, more than half of the patients in our cohort (77/149, 51.7%) reported borderline-to-clinical anxiety symptoms, and 57/149 (38.3%) had borderline depressive symptoms. By the end of NAC, the proportion of patients with clinically significant depression rose from 0/149 (0%) at baseline to 18/149 (12.1%), indicating de novo emergence of clinical depressive symptoms during the treatment course. EORTC global health scores declined by a clinically meaningful ≥10 points in 51/149 patients (34.2%), with a numerically larger burden in luminal B and TNBC than in HER2-positive disease - a difference that did not reach statistical significance (χ²=4.63, df=2, p=0.099) but is consistent with the more intensive cytotoxic profile of regimens used in those subtypes. Our findings parallel those of Browall et al., who documented a significant decline in multiple HRQoL domains during adjuvant chemotherapy in postmenopausal women with breast cancer, with partial recovery only after treatment completion [30]. In a prospective cohort comparing breast cancer survivors with the general population at one year post-diagnosis, Lee et al. showed that HRQoL deficits attributable to cancer treatment persisted well beyond the active treatment phase, particularly in younger women and in those who had received chemotherapy [31]. Our observation that clinically meaningful HRQoL decline affected more than one-third of our cohort by the end of NAC alone, before any post-surgical recovery, aligns with prior literature and emphasizes that the burden of treatment is substantial even within the relatively short window of neoadjuvant therapy.

The observed trend toward differential HADS-A trajectories across subtypes (Kruskal-Wallis H=5.92, df=2, p=0.052), with rising anxiety in HER2-positive patients and declining anxiety in luminal B patients, deserves cautious interpretation - it may reflect the distinct experience of prolonged maintenance HER2-directed therapy beyond surgery, which sustains a treatment-related identity and uncertainty over a longer horizon. That baseline PROs did not predict pCR after adjustment for biological variables is compatible with the body of work suggesting that psychological distress may modulate cancer outcomes through neuroendocrine and immune pathways, since that literature concerns predominantly long-term oncological endpoints, including progression, recurrence, and survival, rather than the short-term, biologically determined endpoint of pathological response [32,33]. Two complementary interpretations of our null finding are warranted. First, the size and design of our cohort may have been underpowered to detect modest independent effects of baseline distress on a binary endpoint such as pCR. Second, and more importantly, the most relevant outcomes for psychological factors are likely longer-term and adherence-mediated, including treatment continuation, persistence of endocrine therapy in the adjuvant phase, late recurrence, and survival, rather than acute tumor shrinkage during cytotoxic therapy. The borderline trend toward higher pCR odds in patients with lower baseline global health (OR: 2.89, 95% CI: 0.99-8.40, p=0.052) should not be over-interpreted. The most biologically plausible reading is that more advanced or more symptomatic tumors are also more proliferative and therefore more chemosensitive, so that the apparent association reflects tumor biology imperfectly captured by clinical staging rather than a true effect of psychological distress on chemosensitivity. Given the small number of events and the borderline statistical significance, this observation remains hypothesis-generating and should not be considered evidence of a distress-mediated effect on pathological response.

The clinical implications of the patient-reported findings are arguably more immediately actionable than those of the biological findings. The high baseline burden of distress, before any cytotoxic exposure, argues for systematic baseline screening for anxiety and depression, using brief, validated instruments such as HADS or PHQ-4, as a standard component of pre-NAC evaluation, with established pathways to psycho-oncology support [34,35]. The de novo emergence of clinical depression during treatment further supports in-treatment rescreening rather than reliance on a single baseline assessment. Such screening is low-cost to implement, although its clinical value depends on the existence of established referral pathways to psycho-oncology services; where such pathways are in place, screening has the potential to identify a substantial subgroup of patients who would benefit from supportive intervention, irrespective of their probability of achieving pCR.

Strengths and limitations

Several limitations should be acknowledged. First, the single-center, retrospective design exposes our analysis to selection and information biases that no statistical adjustment fully eliminates. Patients treated at a tertiary oncology center in Bucharest may differ in socioeconomic profile, access to care, and baseline clinical characteristics from the general Romanian breast cancer population, which limits the generalizability of our findings to other healthcare settings, in particular to public-sector cohorts and to populations treated in the era of routine immune checkpoint blockade in early TNBC. External validation in independent multi-center cohorts is required before our findings can be applied clinically. Second, although the apparent discrimination of our multivariable model was high (AUC: 0.93, bootstrap bias-corrected 0.92), this metric should be interpreted with caution given the cohort size (149 patients, 41 pCR events) and the events-per-variable ratio of approximately eight for the five candidate predictors entered into the model; while bootstrap optimism estimation supports the internal robustness of the model, the absence of external validation precludes any conclusion regarding generalizability, and the possibility of overfitting cannot be excluded. The discrimination metrics should therefore be regarded as exploratory and hypothesis-generating. Third, our sample size, while comparable to many single-institution NAC series, limits power for subgroup analyses, particularly within TNBC and for the detection of subtle interactions between PROs and biological variables. Fourth, we did not collect intermediate PRO measurements during NAC, which would have enabled finer modeling of distress trajectories and the identification of inflection points relevant to psychosocial intervention. Fifth, survival data are not yet mature; the ultimate clinical relevance of biological and PRO variables in our cohort with respect to event-free and overall survival, and particularly with respect to late recurrence in the HR-positive subgroup, will be addressed in a planned long-term follow-up analysis. Finally, biomarker analyses beyond TILs, including serial circulating tumor DNA (ctDNA) dynamics during NAC, residual ctDNA after surgery as a marker of molecular minimal residual disease, and multi-gene expression signatures capturing proliferation, immune, and stromal biology, were beyond the scope of the present analysis. Integration of these biomarkers represents a planned extension of this work, with particular translational relevance for the monitoring of dormant residual disease and late recurrence in hormone receptor-positive breast cancer.

## Conclusions

In a real-world Romanian cohort of women treated with modern NAC for early breast cancer, pCR was independently associated with clinical subtype and stromal tumor-infiltrating lymphocytes, in line with contemporary literature; the predictive model showed high apparent discrimination but requires external validation before any clinical application. Although baseline patient-reported outcomes did not improve prediction of pCR, the high prevalence of pre-treatment anxiety and the de novo emergence of clinical depression during treatment represent a major and largely modifiable clinical burden. Our findings support the routine integration of psychosocial screening, paired with established referral pathways to psycho-oncology services, alongside biological assessment throughout the neoadjuvant care pathway, with longer-term outcomes (event-free survival, distant recurrence-free survival, and late recurrence in hormone receptor-positive disease) as the next frontier of investigation.

## References

[1] Mieog JS, van der Hage JA, van de Velde CJ (2007). Neoadjuvant chemotherapy for operable breast cancer. Br J Surg.

[2] Kaufmann M, von Minckwitz G, Mamounas EP (2012). Recommendations from an international consensus conference on the current status and future of neoadjuvant systemic therapy in primary breast cancer. Ann Surg Oncol.

[3] Liu X, Bergman LE, Boman C, Foukakis T, Matikas A (2025). Long-term outcome for neoadjuvant versus adjuvant chemotherapy in early breast cancer and the prognostic impact of nodal therapy response: a population-based study. Eur J Surg Oncol.

[4] Cortazar P, Zhang L, Untch M (2014). Pathological complete response and long-term clinical benefit in breast cancer: the CTNeoBC pooled analysis. Lancet.

[5] Cortazar P, Geyer CE Jr (2015). Pathological complete response in neoadjuvant treatment of breast cancer. Ann Surg Oncol.

[6] Antonini M, Mattar A, Pereira TM (2025). Pathologic complete response and breast cancer survival post-neoadjuvant chemotherapy: a systematic review and meta-analysis of real-world data. Heliyon.

[7] von Minckwitz G, Untch M, Blohmer JU (2012). Definition and impact of pathologic complete response on prognosis after neoadjuvant chemotherapy in various intrinsic breast cancer subtypes. J Clin Oncol.

[8] Spring LM, Fell G, Arfe A (2020). Pathologic complete response after neoadjuvant chemotherapy and impact on breast cancer recurrence and survival: a comprehensive meta-analysis. Clin Cancer Res.

[9] Houssami N, Macaskill P, von Minckwitz G, Marinovich ML, Mamounas E (2012). Meta-analysis of the association of breast cancer subtype and pathologic complete response to neoadjuvant chemotherapy. Eur J Cancer.

[10] Curigliano G, Burstein HJ, Gnant M (2023). Understanding breast cancer complexity to improve patient outcomes: the St Gallen International Consensus Conference for the primary therapy of individuals with early breast cancer 2023. Ann Oncol.

[11] Salgado R, Denkert C, Demaria S (2015). The evaluation of tumor-infiltrating lymphocytes (TILs) in breast cancer: recommendations by an International TILs Working Group 2014. Ann Oncol.

[12] Denkert C, von Minckwitz G, Darb-Esfahani S (2018). Tumour-infiltrating lymphocytes and prognosis in different subtypes of breast cancer: a pooled analysis of 3771 patients treated with neoadjuvant therapy. Lancet Oncol.

[13] Aaronson NK, Ahmedzai S, Bergman B (1993). The European Organization for Research and Treatment of Cancer QLQ-C30: a quality-of-life instrument for use in international clinical trials in oncology. J Natl Cancer Inst.

[14] Zigmond AS, Snaith RP (1983). The hospital anxiety and depression scale. Acta Psychiatr Scand.

[15] Mehnert A, Hartung TJ, Friedrich M (2018). One in two cancer patients is significantly distressed: prevalence and indicators of distress. Psychooncology.

[16] Bjelland I, Dahl AA, Haug TT, Neckelmann D (2002). The validity of the hospital anxiety and depression scale: an updated literature review. J Psychosom Res.

[17] Amin MB, Greene FL, Edge SB (2017). The eighth edition AJCC Cancer Staging Manual: continuing to build a bridge from a population-based to a more "personalized" approach to cancer staging. CA Cancer J Clin.

[18] Symmans WF, Peintinger F, Hatzis C (2007). Measurement of residual breast cancer burden to predict survival after neoadjuvant chemotherapy. J Clin Oncol.

[19] Snaith RP (2003). The Hospital Anxiety and Depression Scale. Health Qual Life Outcomes.

[20] Osoba D, Rodrigues G, Myles J, Zee B, Pater J (1998). Interpreting the significance of changes in health-related quality-of-life scores. J Clin Oncol.

[21] Harrell FE Jr, Lee KL, Mark DB (1996). Multivariable prognostic models: issues in developing models, evaluating assumptions and adequacy, and measuring and reducing errors. Stat Med.

[22] Hosmer DW, Lemeshow S (1980). Goodness of fit tests for the multiple logistic regression model. Commun Stat Theory Methods.

[23] Loi S, Drubay D, Adams S (2019). Tumor-infiltrating lymphocytes and prognosis: a pooled individual patient analysis of early-stage triple-negative breast cancers. J Clin Oncol.

[24] Schmid P, Cortes J, Pusztai L (2020). Pembrolizumab for early triple-negative breast cancer. N Engl J Med.

[25] Mittendorf EA, Zhang H, Barrios CH (2020). Neoadjuvant atezolizumab in combination with sequential nab-paclitaxel and anthracycline-based chemotherapy versus placebo and chemotherapy in patients with early-stage triple-negative breast cancer (IMpassion031): a randomised, double-blind, phase 3 trial. Lancet.

[26] Loibl S, André F, Bachelot T (2024). Early breast cancer: ESMO Clinical Practice Guideline for diagnosis, treatment and follow-up. Ann Oncol.

[27] Symmans WF, Wei C, Gould R (2017). Long-term prognostic risk after neoadjuvant chemotherapy associated with residual cancer burden and breast cancer subtype. J Clin Oncol.

[28] Pan H, Gray R, Braybrooke J (2017). 20-year risks of breast-cancer recurrence after stopping endocrine therapy at 5 years. N Engl J Med.

[29] Pedersen RN, Esen BÖ, Mellemkjær L (2022). The incidence of breast cancer recurrence 10-32 years after primary diagnosis. J Natl Cancer Inst.

[30] Browall M, Ahlberg K, Karlsson P, Danielson E, Persson LO, Gaston-Johansson F (2008). Health-related quality of life during adjuvant treatment for breast cancer among postmenopausal women. Eur J Oncol Nurs.

[31] Lee ES, Lee MK, Kim SH (2011). Health-related quality of life in survivors with breast cancer 1 year after diagnosis compared with the general population: a prospective cohort study. Ann Surg.

[32] Bortolato B, Hyphantis TN, Valpione S (2017). Depression in cancer: the many biobehavioral pathways driving tumor progression. Cancer Treat Rev.

[33] Lutgendorf SK, Sood AK, Antoni MH (2010). Host factors and cancer progression: biobehavioral signaling pathways and interventions. J Clin Oncol.

[34] Andersen BL, Lacchetti C, Ashing K (2023). Management of anxiety and depression in adult survivors of cancer: ASCO Guideline update. J Clin Oncol.

[35] Howell D, Keller-Olaman S, Oliver TK (2013). A pan-Canadian practice guideline and algorithm: screening, assessment, and supportive care of adults with cancer-related fatigue. Curr Oncol.

